# Visceral to total obesity ratio and severe hydronephrosis are independently associated with prolonged pneumoperitoneum operative time in patients undergoing laparoscopic radical nephroureterectomy for upper tract urothelial carcinoma

**DOI:** 10.1186/s40064-015-1077-5

**Published:** 2015-06-24

**Authors:** Keisuke Shigeta, Eiji Kikuchi, Masayuki Hagiwara, Seiya Hattori, Gou Kaneko, Masanori Hasegawa, Toshikazu Takeda, Masahiro Jinzaki, Hirotaka Akita, Akira Miyajima, Ken Nakagawa, Mototsugu Oya

**Affiliations:** Department of Urology, Keio University School of Medicine, 35 Shinanomachi, Shinjuku-ku, Tokyo, 160-8582 Japan; Department of Diagnostic Radiology, Keio University School of Medicine, Tokyo, Japan

**Keywords:** Visceral obesity, Severe hydronephrosis, Laparoscopic radical nephroureterectomy, Upper tract urothelial carcinoma

## Abstract

**Background:**

Our aim was to evaluate the effect of visceral obesity and impact of severe hydronephrosis on surgical complexity in patients undergoing laparoscopic radical nephroureterectomy (LRNU).

**Methods:**

From January 2000 to December 2013, 169 patients underwent radical nephroureterectomy at our institution. We retrospectively reviewed the medical records of 70 patients who underwent LRNU. We measured total fat area (TFA) and visceral fat area (VFA) at the level of the umbilicus using computed tomography. We defined accumulated visceral fat distribution as a VFA/TFA ratio ≥0.45. Ipsilateral hydronephrosis was graded from 0 to 4 by two uro-radiologists blinded to the clinical outcomes.

**Results:**

Among the 70 patients, VFA/TFA ratio was ≥0.45 in 40 patients (57.1%) and 28 (40.0%) had severe hydronephrosis (grade 3 or greater). Patients with a VFA/TFA ratio ≥0.45 had significantly longer pneumoperitoneum and total operation times compared to their counterparts (p = 0.047 and p = 0.002, respectively). Patients with severe hydronephrosis had significantly longer pneumoperitoneum and total operative times compared to their counterparts (p = 0.006 and p = 0.002, respectively). Multivariate logistic regression analysis showed that a high VFA/TFA and severe hydronephrosis were independent predictive factors for prolonged pneumoperitoneum (p = 0.048, HR = 2.90; p = 0.015, HR = 3.82, respectively) and total operative times (p < 0.001, HR = 18.7; p = 0.003, HR = 10.7; respectively). Other pre-clinical factors such as age, gender, BMI, clinical stage, tumor size, location, laterality, degree of perinephric stranding, and surgical procedure did not affect the operation times.

**Conclusion:**

The present data indicated that the visceral type of adipose accumulation and presence of severe hydronephrosis could provide preoperative information on the degree of technical difficulty associated with LRNU.

**Electronic supplementary material:**

The online version of this article (doi:10.1186/s40064-015-1077-5) contains supplementary material, which is available to authorized users.

## Background

Upper tract urothelial carcinoma (UTUC) is a relatively uncommon disease and accounts for about 5% of all urothelial tumors and 5–10% of all renal tumors (Siegel and Naishadham 2012). UTUC is located more commonly in the renal pelvis than in the ureter, with a ratio of 2–3:1, has a peak incidence in people in their 70s and 80s, and is more prevalent in men than women (Lughezzani et al. 2010; Gandaglia et al. 2014). Open radical nephroureterectomy (ORNU) with excision of a bladder cuff has been the gold standard of treatment for UTUC since its description by Albarran in 1909. Recently, this concept has been shifting to laparoscopic radical nephroureterectomy (LRNU), which has been shown to be as effective as ORNU with respect to oncologic outcomes while resulting in less perioperative morbidity in localized UTUC patients (Adibi et al. 2012). Laparoscopic surgery has several advantages over open surgery, including a faster recovery time with a reduced hospital stay, fewer wound-related complications, and improved cosmetics. Since Clayman et al. (1991) first described the technique of LRNU, it has emerged as an accepted minimally invasive treatment alternative to ORNU (Sugihara et al. 2013).

In laparoscopic abdominal surgeries, the relationship between obesity and prolonged operative time has been reported by several studies (Hagiwara et al. 2012; Beddy et al. 2010). Operation prolongation due to the surgical procedure can be associated with increased risk of complications, such as deep venous thrombosis and pulmonary embolism (Secin et al. 2008). Therefore, it is important for surgeons to have valid information before surgery on exactly which factor would prolong the operation time. Many studies have reported that obesity is thought to be the major factor influencing the degree of technical difficulty encountered during laparoscopic surgery (Hagiwara et al. 2012; Beddy et al. 2010; Hasegawa et al. 2013). In previous studies, body mass index (BMI) was widely used as an indicator of the degree to which a patient is obese, and served as a substitute for an objective parameter of obesity (Hagiwara et al. 2011). However, recent studies have revealed that high BMI does not necessarily predict surgical complexity because the distributions of visceral and subcutaneous adipose tissue differ greatly among individuals. Therefore, the focus of attention has recently shifted to the degree of visceral obesity by measuring visceral fat area (VFA) using computed tomography (CT) for an accurate evaluation of obesity (Ehdaie et al. 2011; Moon et al. 2008; Ioffe et al. 2013; Kobayashi et al. 2002). In laparoscopic nephrectomy, Hagiwara et al. reported that visceral fat accumulation was an independent predictive factor which affected surgical operative time to a greater extent than BMI (Hagiwara et al. 2012). Although there is currently a lot of ongoing discussion about the relationship of visceral obesity and surgical outcomes, the effect of visceral obesity on surgical outcomes in LRNU has not been fully described.

In patients with UTUC, ipsilateral hydronephrosis at diagnosis may be considered a risk factor that will influence surgical complexity during surgery. Although limited information has been reported on the association between hydronephrosis and surgical complexity, severe hydronephrosis may narrow the surgical field and thus hinder access to the renal hilum and vessels, which can have a negative effect on the ability of the surgeon during the surgical procedure (Kurokawa et al. 2002). Moreover, Ito et al. (2011) reported the preoperative hydronephrosis grade is significantly associated with aggressive disease features in UTUC, which can potentially complicate the surgical procedure. Taking these findings into consideration, a delicate maneuver by the surgeon may be needed to retrieve the kidney, dilated renal pelvis, and ureter en bloc with the bladder cuff in order to minimize the risk of tumor dissemination. Therefore, we conducted a retrospective analysis to identify clinical factors that increase the degree of technical difficulty of the surgical procedure for LRNU. Our aim was to examine the effect of visceral obesity and impact of severe hydronephrosis on surgical complexity in patients who underwent LRNU. In the present study, we evaluated the operative time by dividing it into pneumoperitoneum time and bladder cuff excision time and then re-reviewing the videotapes retrospectively.

## Methods

### Patient population

A total of 169 patients diagnosed with UTUC at Keio University Hospital from January 2000 to December 2013 were treated by radical nephroureterectomy. Of these patients, ORNU was performed in 91 and LRNU in 78. We excluded LRNU patients with a history of muscle-invasive urothelial carcinoma of the urinary bladder (n = 5) and those who were treated with neoadjuvant chemotherapy (n = 3). Thus, 70 LRNU patients were finally included in our study population. No patient underwent endoscopic resection prior to LRNU in our population. Basically LRNU was performed for clinical T3 or less UTUCs without positive lymph node or distant metastasis (cTa-3N0M0). Of the 70 patients, 52 (74.3%) were men and 18 (25.7%) were women. The tumor was located in the renal pelvis in 37 (52.9%) patients and the ureter in 33 (47.1%) patients.

This study was approved by the Institutional Review Board of Keio University School of Medicine.

### Surgical procedure

LRNU was performed according to the standard procedure, in other words, extrafascial dissection of the kidney with 2/3 of the length of the ureter resected together under laparoscopic procedure. Under general anesthesia, the patient was placed in the lateral decubitus 60° flank position. LRNU was performed using either a transperitoneal approach or retroperitoneal approach for extirpation of the kidney, and a small iliac incision (Gibson incision) was made to retrieve the kidney and ureter en bloc and to perform resection of the bladder cuff. The laparoscopic procedures were performed with 3 trocars, and 1 trocar was added for liver retraction during right LRNU in the transperitoneal approach. Routine regional lymph node dissection was not performed.

To accurately determine the technical difficulty associated with LRNU, we divided the total procedure into three categories; extirpation of the kidney, bladder cuff excision, and total operation. We then measured the operation time in each group and defined total surgical operative time as the sum of the pneumoperitoneum time and bladder cuff excision time. Pneumoperitoneum time was defined as the time from infusing pressurized CO_2_ gas after port insertion to extirpation of the kidney by retrospectively reviewing the videotapes in each case.

### Assessment of obesity related parameters and hydronephrosis

**Figure 1:**
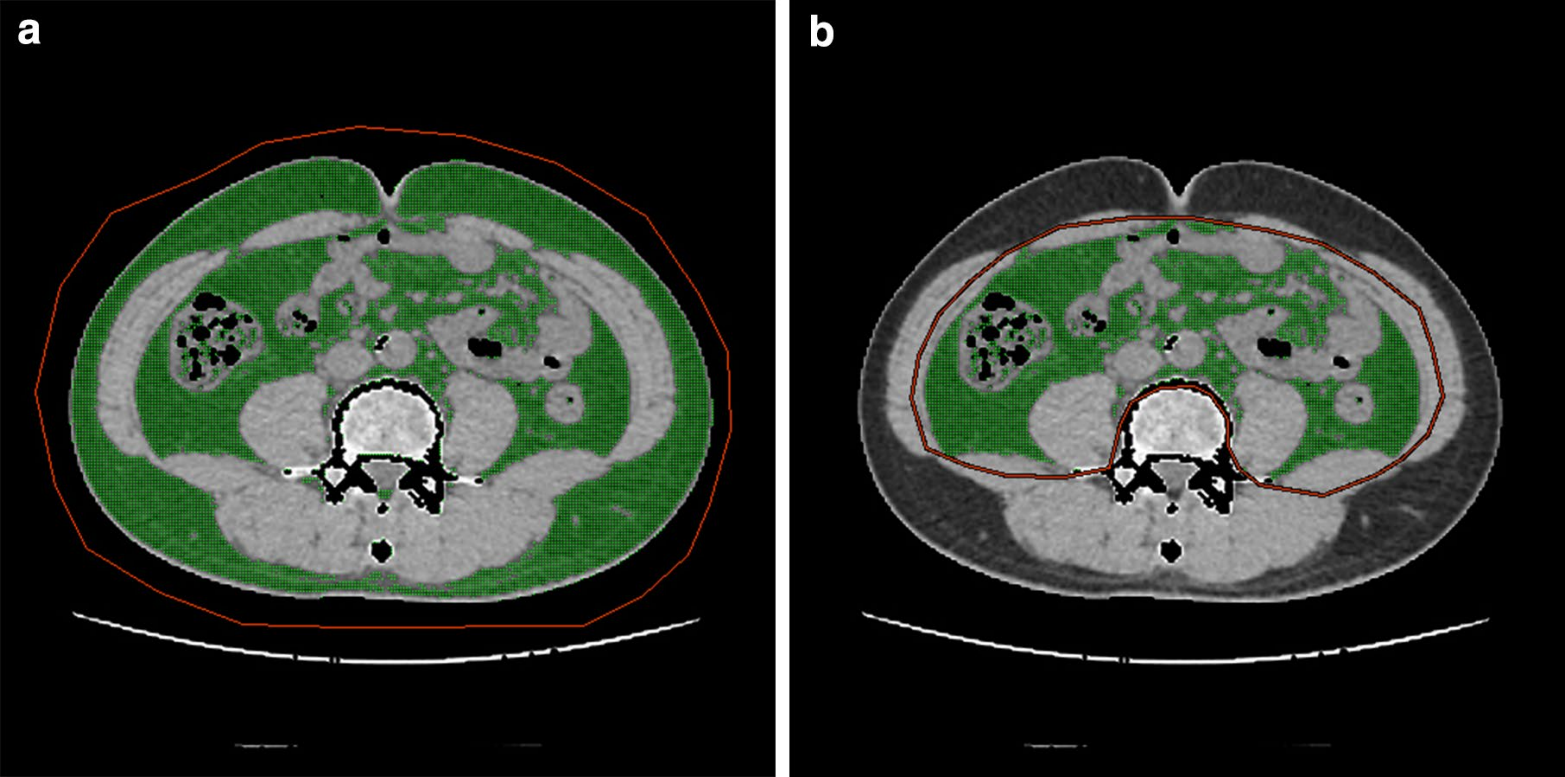
Graphics showing a method for determining the degree of fat distribution on computed tomography. **a** Area inside the *red line* is total fat area (TFA). **b** Area inside the *red line* is visceral fat area (VFA).

Total fat area (TFA), visceral fat area (VFA), and subcutaneous fat area (SFA) were measured at the level of the umbilicus using CT according to a procedure described and validated previously (Kobayashi et al. 2002; Seidell et al. 1987). The tomographic attenuation of the adipose tissue was defined to be between −50 and −150 Hounsfield Units. As shown in Figure 1, the border of the intra-abdominal cavity was outlined on the CT image, and TFA and VFA were then quantified using standard software (Advantage Work Station). The SFA was calculated by subtracting VFA from TFA. In order to estimate the degree of proportional adipose tissue distribution, we developed the VFA/TFA ratio calculated on the basis of measured data, as a practical and standard parameter for the type of obesity using CT scan. Two genitourinary radiologists completed all the measurements and were blinded to the clinical details of the subjects. BMI was also calculated for all patients.

For grading the degree of ipsilateral hydronephrosis, preoperative CT images and/or MRI images were obtained by two genitourinary radiologists who were blinded to the clinical details. Ipsilateral hydronephrosis was graded from 0 to 4 according to the classification of Cho et al. (2007). Cases without calyx or pelvic dilation were classified as grade 0, cases with pelvic dilation only were classified as grade 1, cases with mild calyx dilation were classified as grade 2, cases with severe calyx dilation were classified as grade 3, and cases with calyx dilation accompanied by renal parenchyma atrophy were classified as grade 4 hydronephrosis (Ito et al. 2011). Furthermore, we reviewed the degree of perinephric stranding, defined as linear area of soft tissue attenuation in the perinephric space, for each kidney on CT. Stranding was graded as 0 (no stranding), grade 1 (thin rimlike mild stranding), and grade 2 (diffuse, thick-banded severe stranding) as previously used and described in the literature (Kim et al. 2013; Davidiuk et al. 2014).

### Statistical analysis

All variables are expressed as the mean ± standard deviation. Associations between clinical factors and the related operative parameters were analyzed using the Chi square test and Mann–Whitney U test for categorical and continuous variables, respectively. By considering the mean amount of the variables, we defined VFA/TFA ratio ≥0.45 as a dichotomous variable for visceral adipose accumulated group. Similarly, the mean of each operating time (pneumoperitoneum, bladder cuff excision, and total operative time) was used as a threshold value in order to discriminate between short and long operative times. We chose mean operative time as a cut off value discriminating between short and long operative times since previous studies adopted it as a clinically relevant value (Hagiwara et al. 2011; Hattori et al. 2014). Univariate and multivariate logistic regression analyses were performed to identify independent predictors for prolonged operative time. To identify the predictive factors for surgical complexity during LRNU, we included age (≥70 vs. <70), gender, BMI (≥25 kg/m^2^ vs. <25), clinical stage (stage 3 vs. <3), laterality, tumor location (renal pelvis vs. ureter), tumor size (diameter ≥20 mm vs. <20), surgical procedure (retroperitoneal vs. transperitoneal), perinephric stranding (grade ≥1 vs. grade 0), VFA/TFA (≥0.45 vs. <0.45), and hydronephrosis grade (grade ≥3, vs. grade <3). A 2-sided P value <0.05 was considered to be significant. All statistical analyses were performed using the SPSS program, version 20.0. (SPSS Inc, Chicago, IL, USA)

## Results

### Patient characteristics

A total of 70 patients who underwent LRNU were identified during the study period. Among these 70 patients, 28 (40%) underwent LRNU by a transperitoneal approach and 42 (60%) by a retroperitoneal approach. The mean age was 69.2 ± 10.4. In our study group, 45 (64.3%) were classified as having a healthy body weight (BMI <25.0 kg/m^2^), 22 (31.4%) as overweight (25.0–29.9 kg/m^2^), and 3 (4.3%) as obese (>30.0 kg/m^2^) according to the World Health Organization classification. The VFA/TFA ratio was calculated to demonstrate the distribution of adipose tissue. The mean BMI, TFA, VFA, SFA, and VFA/TFA ratio were 23.6 ± 3.42 kg/m^2^, 269.3 ± 102.4 cm^2^, 121.3 ± 56.7 cm^2^, 148.0 ± 45.7 cm^2^, and 0.445 ± 0.130, respectively (Additional file 1: Table S1). According to the imaging studies, 43 (61.4%) patients had ipsilateral hydronephrosis preoperatively. The number of Grade 1–4 patients were as follows; grade 1: 5 (7.1%), grade 2: 10 (14.3%), grade 3: 14 (20%), and grade 4: 14 (20%). Severe hydronephrosis (≥grade 3) was identified in 18.9% of patients with tumors in the renal pelvis and in 68.4% in the lower ureter. 35 (50%) patients had no perinephric stranding while 35 (50%) patients had mild to severe perinephric stranding. The mean pneumoperitoneum time, bladder cuff excision time, total operative time, and total blood loss were 162.1 ± 63.4 min, 160.4 ± 92.4 min, 323.7 ± 113 min, and 263.4 ± 565.6 ml, respectively.

### Association between the parameters for obesity, hydronephrosis and surgical operation time

The associations of BMI, VFA, VFA/TFA ratio, degree of hydronephrosis, and surgical operative time are shown in Additional file 2: Table S2.

Univariate analysis indicated that BMI ≥25 was not associated with the prolongation of any surgical operative time in LRNU. VFA ≥100 cm^2^ was associated with the prolongation of total operative time (p = 0.016), but no significant association was found between pneumoperitoneum time and VFA. The VFA/TFA ≥0.45 was significantly associated with the prolongation of pneumoperitoneum and total operative time compared to their counterparts (p = 0.047 and p = 0.002, respectively). Furthermore, severe hydronephrosis (≥grade 3) was also significantly associated with the prolongation of pneumoperitoneum and total operative time compared to their counterparts (p = 0.006 and p = 0.002, respectively). No parameter related to obesity or degree of hydronephrosis was associated with the bladder cuff excision time.

### Predictive factors for identifying prolongation of surgical operation time

Univariate and multivariate logistic regression analysis were performed to identify indicators that prolong the surgical outcome (Additional file 3: Table S3). Univariate regression analysis showed that severe hydronephrosis (≥grade 3) resulted in the prolongation of pneumoperitoneum time (p = 0.022). On the other hand, ureteral tumor (p = 0.031) and VFA/TFA ≥0.45 (p = 0.041) were significant factors for bladder cuff excision prolongation. Furthermore, ureteral tumor (p = 0.017), VFA/TFA ≥0.45 (p < 0.001), and severe hydronephrosis ≥grade 3 (p = 0.010) were significant factors for total operation prolongation. Multivariate analysis using logistic regression demonstrated that VFA/TFA ≥0.45 (p = 0.048) and severe hydronephrosis ≥grade 3 (p = 0.015) were independent predictive factors for prolonged pneumoperitoneum time. Ureteral tumor (p = 0.034) and VFA/TFA ≥0.45 (p = 0.047) were significant predictive factors for prolonged bladder cuff operation. VFA/TFA ≥0.45 (p < 0.001) and severe hydronephrosis (p = 0.003) were identified as independent predictive factors for prolonged total operative time.

## Discussion

In the present study, we demonstrated that visceral fat accumulation and severe hydronephrosis (≥grade 3) had a greater effect on prolonged pneumoperitoneum time and total operation time in LRNU. To the best of our knowledge, this is the first report to demonstrate an association of visceral obesity and hydronephrosis with respect to surgical complexity in LRNU.

Obesity is a common and growing problem in industrialized countries (Bray 2002). Many previous reports have used BMI as an indicator of the degree to which a patient is overweight, and BMI was often used as an indicator of obesity (Parker et al. 2008). However, recent studies indicate that BMI does not always accurately reflect the various types of obesity because it does not actually indicate the distribution of adipose tissue per individual. Therefore, leaders in minimally invasive surgery have begun to realize the limitations of using BMI as an objective parameter of obesity, and their attention has been shifting to the degree of visceral fat of the patient. Previous studies have showed that minimally invasive surgery is difficult to perform in visceral obese patients due to the limited visualization of the surgical field and the presence of cumbersome visceral fat tissue, which result in increased complexity exposing the main vessels (Makino et al. 2010). Ioffe et al. (2013) also emphasized that excess visceral fat in Gerota’s fascia can extend medially, obscuring the origin of the renal vessels, necessitating a more cumbersome and perilous dissection and requiring excess lateral retraction during laparoscopic or robotic surgery for partial nephrectomy. Furthermore, several studies on minimally invasive oncologic procedures have found increased visceral fat to be a risk factor for worse perioperative outcomes, such as increased blood loss and complication rates (Seki et al. 2007). Based on these arguments, we hypothesized that high VFA can theoretically increase the surgical complexity by reducing the effective operative field and visually blocking anatomic structures.

To determine the fat volume in the body, measuring VFA from a single CT scan obtained at the level of the umbilicus was found to be closely correlated with the total volume of visceral fat, rather than BMI (Seidell et al. 1987). Thus, VFA ≥100 cm^2^ was adopted as one diagnostic criteria for visceral obesity (New criteria for ‘obesity disease’ in Japan 2002). Since this criterion was established to predict metabolic disease and obesity-related disorders, Hasegawa et al. reported the correlation of visceral fat and prolongation of operative time in laparoscopic adrenalectomy by adopting the VFA/TFA ratio (Hasegawa et al. 2013). Therefore, we also focused on the distribution of adipose tissue between TFAs and VFAs, which can be measured by the VFA/TFA ratio. Multivariate analysis showed that VFA/TFA ≥0.45 was a significant risk factor for a prolonged total operative time (p < 0.001, HR = 18.7). We also observed that visceral obesity significantly prolonged the pneumoperitoneum time (p = 0.048, HR = 2.90). This result suggests that the level of visceral fat can have a significant influence, especially on the degree of difficulty of the laparoscopic procedure, since pneumoperitoneum time excludes the time of placement of ports and represents the pure effect of visceral fat. In contrast, a high BMI (≥25 kg/m^2^) showed no relationship with any surgical operative time. These results indicate that although obesity could cause the prolongation of operating time, visceral obesity may have a stronger influence and may be a better index than BMI for predicting surgical operating time in LRNU.

Our study also demonstrated that ipsilateral hydronephrosis (≥grade 3) detected by preoperative CT independently predicts the surgical complexity in LRNU. The presence of grade 3 or greater hydronephrosis independently prolonged the pneumoperitoneum time (p = 0.015, HR = 3.82) and total operative time (p = 0.003, HR = 10.7). Previous studies, however, have provided very little information on the relationship between hydronephrosis and surgical complexity. Kurokawa et al. reported their experience of LRNU for dysplastic kidney in four children and described the operative difficulty of these cases with hydronephrosis and hydroureter due to surrounding tissue adhesion of the ureter (Kurokawa et al. 2002). Other authors have also described the operative difficulty associated with laparoscopic nephrectomy for giant hydronephrosis due to the massive size of the renal pelvis and ureter, altered anatomic relationships, and chronic recurrent infections (Hemal et al. 1999; Harper et al. 2007). Bosoteanu et al. reported on the pathologic and morphological correlations in congenital hydronephrosis. According to their findings, congenital hydronephrosis leads to a complex change in the pyelic wall, such as epithelial atrophy with elastic tissue loss caused by chronic inflammatory infiltrate and tissue fibrosis (Bosoteanu et al. 2011). Although these references are based on pediatric diseases, we assumed that severe hydronephrosis leads to dilatation of the renal pelvis and ureter, which results in a fragile and thin pelvic/ureteric wall with adjacent tissue adhesion caused by chronic inflammation. Therefore, surgeons are required to perform a careful maneuver to avoid damaging the ureteral wall, which could result in ureteral rupture and tumor dissemination. As a result, a longer operative time would be inevitable to perform an accurate and safe operation.

The conventional surgical approach for UTUC was for many decades open surgery. However, since discussions resulted in similar oncological outcomes between ORNU and LRNU in many studies, the demand for laparoscopy seems to be continually increasing. As the prevalence of laparoscopic procedures continue to rise, a method for objectively predicting the technical difficulty in LRNU is also desperately needed. Since preoperative CT images are part of the routine assessment used at diagnosis in UTUC patients, the measurement of VFA/TFA and grading ipsilateral hydronephrosis are suitable for predicting surgical operative time because they do not require additional examination. Complicated surgical cases are not suitable, especially for young surgeons performing laparoscopic surgery for UTUC. We believe that our study provides valuable information for surgeons to ensure safe operations by considering the most appropriate surgical strategy preoperatively.

This study has several limitations. It was performed in a retrospective manner and in a limited number of patients. Also, we only included those who underwent LRNU and excluded patients who underwent ORNU. There was also inherent bias since the study was conducted at a single institution. However, all imaging studies were re-reviewed by dedicated genitourinary radiologists to minimize bias, which enabled us to achieve accurate measurements and consistent results.

## Conclusion

We have demonstrated that visceral adipose accumulation and severe hydronephrosis predict the surgical complexity of UTUC patients who are undergoing LRNU. The present study provides useful information for predicting surgical difficulty, and selecting the most suitable surgeon preoperatively.

## Additional files

Additional file 1:
**Table S1.** Patient Characteristics and Demographics. Additional file 2:
**Table S2.** Relationship of BMI, VFA, VFA/TFA, degree of hydronephrosis and surgical operative times. Additional file 3:
**Table S3.** Univariate and multivariate analyses on factors affected prolonged pneumoperitoneum time, bladder cuff excision time, and total operative time.
